# Prevalence and Correlates of Elopement in a Nationally Representative Sample of Children with Developmental Disabilities in the United States

**DOI:** 10.1371/journal.pone.0148337

**Published:** 2016-02-04

**Authors:** Bridget Kiely, Talia R. Migdal, Sujit Vettam, Andrew Adesman

**Affiliations:** Division of Developmental and Behavioral Pediatrics, Department of Pediatrics, Steven and Alexandra Cohen Children's Medical Center of New York, New Hyde Park, New York, United States of America; IRCCS E. Medea, ITALY

## Abstract

Despite increased awareness and concern about children with developmental disabilities wandering away from adult supervision, there is a paucity of research about elopement. This is the first study to examine and report the prevalence and correlates of elopement in a nationally representative sample of school-age children in the United States with an autism spectrum disorder (ASD) and/or cognitive impairment. Data were obtained from the CDC's "Pathways" Survey, a follow-up telephone survey of the parents of 4,032 children with a developmental condition. 3,518 children that had ASD, intellectual disability (ID), and/or developmental delay (DD) at the time of survey administration were included for analysis. Children were divided into three condition groups: ASD-only; ID/DD-only; ASD+ID/DD. Logistic regression analyses were used to compare the prevalence of elopement and rates of preventive measure use (barriers and/or electronic devices) across condition groups, and to examine the clinical and demographic correlates of elopement. T-tests were also performed to compare scores on the Children's Social Behavior Questionnaire (CSBQ) between wanderers and non-wanderers. Overall, 26.7% of children had reportedly eloped within the previous year, most commonly from public places. Children with ASD-only and ASD+ID/DD were more likely to have eloped than those with ID/DD-only. Across all groups, wanderers scored higher than non-wanderers on five out of six CSBQ subscales; they were more likely not to realize when there is danger, to have difficulty distinguishing between strangers and familiar people, to show sudden mood changes, to over-react to everything/everyone, to get angry quickly, to get lost easily, and to panic in new situations or if change occurs. Even after controlling for elopement history, parents of children in the ASD+ID/DD group were more likely than those in the other condition groups to report using physical or electronic measures to prevent wandering.

## Introduction

As the prevalence of developmental disabilities in the United States continues to rise, there is a need to better understand the behaviors that may compromise the safety and well-being of this population [1]. In recent years, several high-profile incidents have drawn attention to wandering, or elopement, as a potential cause of injury in children with developmental disabilities. Numerous children with autism spectrum disorders (ASDs) have reportedly died or sustained serious injuries due to vehicular collisions, drowning, and other accidents that occurred after they wandered away from their caregivers [2]. Elopement may place a significant burden on affected families by causing anxiety and increasing the need for monitoring and supervision. In recognition of the seriousness of this problem, a number of advocacy organizations, including Autism Speaks and the National Autism Association, have created resources designed to educate caregivers, first responders, and others about how to prevent and respond to incidents of elopement [3, 4]. Nonetheless, there is a dearth of published research on elopement in children with developmental disabilities, and little is known about the prevalence and correlates of wandering behaviors in this population [2].

In the largest published study on this subject to date, Anderson et. al. surveyed 1,218 families of children with ASDs drawn from the Interactive Autism Network (IAN) [5]. They found that 49% of the sample had attempted to elope since age four. Analysis of the clinical correlates of wandering showed that elopers, compared to those that had not attempted to elope, tended to have higher scores on the Social Responsiveness Scale (SRS), and lower intellectual and communication developmental quotients. Elopement was also found to have serious consequences for affected families, with 62% of the parents of elopers indicating that concerns about wandering had prevented them from enjoying or attending activities outside of the home. However, given that this study’s sample was composed of voluntary participants from the IAN, it is not clear whether these findings can be generalized to the entire U.S. population of children with ASDs. Moreover, by focusing exclusively on individuals with ASDs, this study was unable to assess the potential impact of elopement on children with other developmental conditions.

Information about the clinical and demographic characteristics of children who elope is needed to guide future efforts to address this troubling issue. The aim of this study was to examine the prevalence and correlates of elopement in a nationally representative sample of U.S. children with developmental disabilities, including ASDs, intellectual disability (ID), and developmental delay (DD).

## Methods

This study, as a secondary analysis of a publicly available, de-identified data set, was determined by the Institutional Review Board of the North Shore-LIJ Health System to be "IRB exempt.”

Data were obtained from the 2011 Survey of Pathways to Diagnosis and Services (“Pathways”), a cross sectional, nationally representative survey of the parents and guardians of children with special health care needs (CSHCN) ages 6–17 with a current or past parent-reported diagnosis of ASD, ID, and/or DD. The Pathways survey was conducted by the CDC’s National Center for Health Statistics (NCHS), State and Local Area Integrated Telephone Survey (SLAITS) program. This survey was a follow-up to the 2009–2010 National Survey of Children with Special Health Care Needs (NS-CSHCN). Within this sample, 7,572 participants were identified as eligible for Pathways, and 6,090 were randomly chosen for follow-up. Among those that were successfully contacted and confirmed as continuing to meet the Pathways eligibility criteria, telephone interviews were completed for 4,032 respondents. A subset of these participants agreed to complete an additional written survey, called the Self-Administered Questionnaire (SAQ), which was composed of questions from the Strengths and Difficulties Questionnaire (SDQ) and the Children’s Social Behavior Questionnaire (CSBQ). In total, 2,988 SAQs were completed and returned [6].

Elopement was assessed based on four questions about the child’s history of wandering from various locations within the previous twelve months. Respondents were asked to indicate whether their child had wandered off or become lost from their own home; from “someone else’s home”; from a structured program such as “school, day care, or summer camp”; or from “a store, restaurant, playground, campsite, or any other public place.” Responses were coded as “yes,” “no,” “don’t know,” or “refused.” The prevalence of elopement was calculated as the percent of respondents that answered “yes” to at least one of these questions. A fifth, “any home” category was also constructed to include all children who had wandered away from their own home and/or someone else’s home.

Analyses were limited to respondents who indicated that their child currently had ASD, ID, and/or DD. Children were assigned to one of three mutually-exclusive condition groups: “ASD-only” (those with current parent-reported ASD without ID or DD); “ASD + ID/DD” (those with current parent-reported ASD and ID and/or DD); and “ID/DD-only” (those with current parent-reported ID and/or DD, without ASD).

The prevalence of elopement within the previous 12 months was calculated separately for the ASD-only, ASD+ID/DD, and ID/DD-only groups. Logistic regression analyses were used to compare the odds of elopement across these groups, and to examine associations between elopement and several demographic factors: age, sex, income, race, ethnicity, and household education level.

To delineate the clinical correlates of wandering, responses to questions from the CSBQ were analyzed for their association with elopement. The CSBQ is a 49-item questionnaire designed to assess a range of symptoms across the Pervasive Developmental Disorder (PDD) spectrum, including milder manifestations [7]. Questions from the CSBQ are grouped into six subscales: “stereotyped behavior” (8 items), “reduced contact and social interest” (12 items), “orientation problems” (8 items), “difficulties in understanding social information” (7 items), “resistance to change” (3 items), and “behavior/emotions not optimally tuned to the situation” (11 items). Responses to each question are scored on a three-point scale; a response of “0” indicates that the difficulty “does not apply”; “1” indicates that it “sometimes or somewhat applies”; and “2” indicates that it “clearly or often applies.” Scores from individual items are then summed to yield subscale scores. The CSBQ has been shown to have good internal consistency, inter-rater reliability, and test-retest reliability, and has been validated for use in individuals with cognitive impairment [7, 8]. Although the criterion-related validity of the CSBQ has also been shown to be good, it differs from a number of other instruments for assessing ASD in that it is not intended to be used for the purposes of diagnostic classification. Rather, the CSBQ reflects a dimensional view of the autism spectrum and is optimal for use in characterizing the pattern and severity of behaviors associated with ASDs [6, 7, 8]. For this reason, the CSBQ represents an ideal tool for examining the association between elopement and the degree of ASD symptomatology—even among children with “sub-threshold” symptoms that do not meet the criteria for a categorical ASD diagnosis. For the present analysis, t-tests were used to compare mean scores on each subscale between wanderers and non-wanderers in each of the three condition groups.

Fifteen items from the CSBQ were identified as being exceptionally relevant to the safety of children who wander, and were thus analyzed individually. These questions focus on the child’s tendency to panic or become angry, their ability to deal with new or unexpected situations, and their awareness of danger, among other qualities. For each of these items, logistic regression analysis was used to compare the odds of indicating that the behavior “clearly or often applies” between wanderers and non-wanderers, after controlling for demographic factors (age, sex, race, ethnicity, household income, and household education level).

A final set of analyses characterized the use of wandering prevention measures in this sample. Use of preventive measures was assessed based on two questions. Respondents were asked to indicate whether they had ever 1) added “fences, gates, locks, alarms, or other barriers” to their homes to avert elopement; and, 2) utilized an electronic tracking device to prevent their child from becoming lost. Logistic regression analyses were performed to compare the odds of preventive measure use across the three condition groups (ASD-only, ASD+ID/DD, and ID/DD-only), and to determine whether a history of elopement within the previous year was associated with higher odds of preventive measure use.

All analyses were performed using SAS SURVEY procedures available in SAS version 9.04 software. Appropriate weights were applied in order to account for the complex sample design of the Pathways survey. Significance was set at the p < 0.05 level; all reported p-values are two-tailed.

## Results

This study utilized data from a nationally representative sample of 4,032 parents and guardians of CSHCN between the ages of 6 and 17 who were reported to have ever been diagnosed with ASD, ID, and/or DD. Once the analysis was restricted to those that currently had at least one of these conditions, the sample was composed of 3,518 participants. At the time of survey administration, 492 had ASD-only; 924 had ASD plus ID and/or DD; and 2,085 had ID and/or DD without ASD. Seventeen participants were not assigned to any of the three condition groups because their parents indicated that they did not know if the child currently had one or more of the developmental conditions (ASD, ID, or DD). Weighted frequencies are reported for all analyses. Comparisons of the demographic characteristics of the three groups are shown in Table 1.

**Table 1:** Demographic Differences among Condition Groups.

Characteristic	ASD-only (% ± SE)	ASD + ID/DD (% ± SE)	ID/DD-only (% ± SE)	p-value	Combined Groups (% ± SE)
Age 6–11 years	53.4 ± 4.0	55.5 ± 2.8	48.2 ± 2.2	0.087	50.6 ± 1.6
Male	88.1 ± 2.2	79.0 ± 2.4	62.8 ± 2.1	<0.001*	69.8 ± 1.5
Non-Hispanic	86.3 ± 3.7	86.8 ± 2.1	86.3 ± 1.8	0.987	86.5 ± 1.3
White	82.4 ± 3.2	68.7 ± 2.8	67.3 ± 2.3	0.003*	69.4 ± 1.7
Non-Hispanic white	71.3 ± 4.2	62.7 ± 2.8	59.4 ± 2.3	0.047*	61.6 ± 1.7
Household income (≤100% FPL)	10.1 ± 2.8	20.4 ± 2.6	32.0 ± 2.3	<0.001*	26.5 ± 1.6
Household education: HS or less	13.8 ± 3.0	27.9 ± 2.9	37.4 ± 2.3	<0.001*	32.3 ± 1.7

**Notes:**

*denotes significance; demographic characteristics were compared across groups using Rao-Scott chi-square tests;

FPL = federal poverty level; HS = high school.

### Prevalence of Elopement

Among all children with a current developmental condition (ASD, ID, and/or DD), the overall rate of elopement within the previous 12 months was 26.7%. Across all groups, public places were the most common location from which elopement reportedly occurred (Table 2).

**Table 2:** Prevalence of Elopement by Location and Condition Group.

Location	ASD-only (% ± SE)	ASD + ID/DD (% ± SE)	ID/DD-only (% ± SE)	Entire Cohort (% ± SE)
Any location	32.7 ± 4.1	34.6 ± 2. 8	22. 7 ± 2.1	26.7 ± 1.6
Own home	8.3 ± 2.3	11.7 ± 1.9	9.6 ± 1.9	9.9 ± 1.3
Another home	4.8 ± 1.7	6.3 ± 1.4	4.3 ± 1.0	4.8 ± 0.7
Any home	11.7 ± 2.7	14.2 ± 2.0	11.1 ± 1.9	11.9 ± 1.4
Structured program	10.2 ± 2.4	10.3 ± 1.9	4.1 ± 0.7	6.3 ± 0.7
Public place	22.5 ± 3.7	25.1 ± 2.7	15.5 ± 1.6	18.6 ± 1.3

**Notes:** All estimates are weighted. The “any home” category refers to children who wandered from their own home and/or another person’s home.

Logistic regression analyses indicated that the odds of elopement differed significantly according to condition group (Table 3). Compared to the ID/DD-only group, the odds of elopement were higher for the ASD-only group (OR = 1.7, 95% CI: 1.1–2.6) and the ASD+ID/DD-group (OR = 1.8, 95% CI: 1.3–2.5). Elopement rates did not differ significantly between the ASD-only and ASD+ID/DD groups (OR = 0.9, 95% CI: 0.6–1.4). After adjusting for several demographic factors (age, sex, income, race, ethnicity, and household education level) the difference between the ASD + ID/DD and the ID/DD-only groups remained significant (aOR = 1.6, 95% CI: 1.1–2.4), although the difference between the ASD-only and the ID/DD-only groups did not (aOR = 1.5, 95% CI: 0.9–2.3).

**Table 3:** Between-group Comparisons of the Odds of Any Elopement within the Previous 12 Months.

Condition Groups	OR (95% CI)	aOR^¥^ (95% CI)
ASD-only vs. ID/DD-only (ref)	1.7* (1.1–2.6)	1.5 (0.9–2.3)
ASD + ID/DD vs. ID/DD-only (ref)	1.8* (1.3–2.5)	1.6* (1.1–2.4)
ASD-only vs. ASD + ID/DD (ref)	0.9 (0.6–1.4)	0.9 (0.6–1.4)

**Notes:**

*denotes significance;

^**¥**^ odds ratios adjusted for age, sex, income, race, ethnicity, and household education;

ref = reference group.

### Demographic Correlates

Logistic regression analyses showed that children who were between the ages of 6 and 11 were more likely to have eloped than those that were 12–17 at the time of the Pathways interview (Table 4). In the ASD+ID/DD group, the odds of elopement were higher for children from households in which the highest level of educational attainment was high school or less. However, education was not associated with wandering in the other two groups. No association was identified between elopement and household income, sex, or race/ethnicity in any group.

**Table 4:** Demographic Characteristics Associated with Elopement.

Characteristic	ASD-only		ASD+ID/DD		ID/DD-only	
	Percent that have wandered (± SE)	aOR (95% CI)	Percent that have wandered (± SE)	aOR (95% CI)	Percent that have wandered (± SE)	aOR (95% CI)
**Age**						
6–11	41.6 ± 5.7	2.4 (1.2–4.9)*	40.6 ± 4.0	1.7 (1.0–2.9)*	30.4 ± 3.6	2.5 (1.6–4.0)*
12–17	22.5 ± 5.0	Ref	27.1 ± 3.8	Ref	15.6 ± 2.1	Ref
**Sex**						
Male	32.5 ± 4.4	1.0 (0.4–2.9)	37.2 ± 3.2	1.7 (0.9–3.2)	22.3 ± 3.0	0.9 (0.5–1.4)
Female	34.2 ± 9.1	Ref	24.9 ± 4.8	Ref	23.6 ± 2.9	Ref
**Income**						
≤ 100% FPL	36.9 ± 9.8	1.4 (0.3–6.4)	36.4 ± 6.9	1.2 (0.6–2.4)	26.3 ± 4.4	1.5 (0.9–2.4)
>100% FPL	30.8 ± 4.4	Ref	33.1 ± 3.2	Ref	22.2 ± 2.7	Ref
**Education**						
≤ HS	21.9 ± 11.1	0.6 (0.2–2.4)	48.4 ± 6.8	2.1 (1.1–4.0)*	22.7 ± 3.7	0.8 (0.5–1.3)
> HS	34.5 ± 4.4	Ref	29.3 ± 2.8	Ref	23.0 ± 2.7	Ref
**Race/Ethnicity**						
Hispanic	48.8 ± 13.3	2.0 (0.6–7.3)	39.5 ± 10.2	1.0 (0.5–2.2)	26.1 ± 6.6	1.4 (0.7–2.9)
Black, non-Hisp.	56.8 ± 15.5	3.0 (0.9–10.2)	23.7 ± 8.2	0.5 (0.2–1.3)	25.0 ± 4.8	1.1 (0.6–2.2)
White, non-Hisp.	29.2 ± 4.1	Ref	36.5 ± 3.4	Ref	19.5 ± 2.0	Ref

**Notes:**

*denotes significance. Logistic regression analyses were used to examine the demographic correlates of elopement in each of the three condition groups;

ref = reference group.

### Clinical Correlates

Across all three groups, wanderers scored significantly higher than non-wanderers on five of the CSBQ subscales. These included the “orientation problems” subscale, the “reduced contact and social interest” subscale, the “difficulties in understanding social information” subscale, the “stereotyped behavior” subscale, and the “behavior/emotions not optimally tuned to the situation” subscale (Table 5; p < 0.05 for all). Wanderers scored higher than non-wanderers on the sixth CSBQ subscale (“resistance to change”) in the ASD+ID/DD (p = 0.018) and the ID/DD-only (p < 0.001) groups; however, within the ASD-only group, scores on this subscale did not differ significantly between wanderers and non-wanderers. When the entire sample was pooled, wanderers scored higher than non-wanderers on all CSBQ subscales.

**Table 5:** Comparison of CSBQ Subscale Scores between Wanderers and Non-wanderers.

Subscale	ASD-only (mean score ± SE)			ASD + ID/DD (mean score ± SE)			ID/DD-only (mean score ± SE)			Entire Sample (mean score ± SE)		
	W	Non-W	p-value	W	Non-W	p-value	W	Non-W	p-value	W	Non-W	p-value
1. Behavior/ emotions not optimally tuned to the situation	13.2 ±0.9	9.9 ±0.6	<0.001*	13.2 ±0.6	10.2 ±0.4	<0.001*	12.1 ±0.5	8.3 ±0.3	<0.001*	12.6 ±0.4	8.9 ±0.2	<0.001*
2. Reduced contact and social interest	9.4 ±0.9	7.2 ±0.6	<0.001*	11.9 ±0.5	8.2 ±0.4	<0.001*	6.7 ±0.5	4.8 ±0.3	0.002*	8.8 ±0.4	5.8 ±0.2	<0.001*
3. Orientation problems	8.7 ±0.7	4.6 ±0.3	<0.001*	10.2 ±0.4	7.3 ±0.3	<0.001*	8.0 ±0.5	5.3 ±0.2	<0.001*	8.9 ±0.3	5.6 ±0.2	<0.001*
4. Difficulties in understanding social information	9.6 ±0.3	6.6 ±0.3	<0.001*	9.7 ±0.4	8.2 ±0.3	0.002*	8.0 ±0.3	5.7 ±0.2	<0.001*	8.8 ±0.2	6.4 ±0.1	<0.001*
5. Stereotyped behavior	6.7 ±0.8	4.6 ±0.4	0.004*	9.4 ±0.4	6.3 ±0.3	<0.001*	4.9 ±0.3	3.1 ±0.2	<0.001*	6.7 ±0.3	4.0 ±0.2	<0.001*
6. Resistance to change	2.9 ±0.3	2.7 ±0.1	0.389	3.6 ±0.2	3.0 ±0.2	0.018*	2.6 ±0.2	1.8 ±0.1	<0.001*	3.0 ±0.1	2.2 ±0.1	<0.001*

**Notes:**

*denotes significance; mean scores on the six subscales of the CSBQ were compared between wanderers (W) and non-wanderers (Non-W) in the three condition groups and in the sample as a whole.

Wanderers and non-wanderers also differed in their responses to a number of individual CSBQ items (Table 6). Among all children with current parent-reported ASD, ID, and/or DD, wanderers were more likely than non-wanderers to show sudden mood changes (aOR = 2.3, 95% CI: 1.6–3.3); to over-react to everything and everyone (aOR = 1.9, 95% CI: 1.3–2.9); to get angry quickly (aOR = 2.1, 95% CI: 1.5–3.0); not to realize when there is danger (aOR = 2.2, 95% CI: 1.5–3.4); to have difficulty distinguishing between strangers and familiar people (aOR = 2.4, 95% CI: 1.5–3.8); to be disobedient (aOR = 2.1, 95% CI: 1.4–3.2); to panic in new situations or if change occurs (aOR = 2.4, 95% CI: 1.6–3.5); to remain clammed up in new situations or if change occurs (aOR = 2.6, 95% CI: 1.7–4.1); and to get lost easily (aOR = 5.2, 95% CI: 3.3–8.4).

**Table 6:** Comparison of the Behavioral Characteristics of Wanderers and Non-wanderers.

Characteristic (CSBQ item)	Wanderers, ASD ± ID ± DD (% ± SE)	Non-Wanderers, ASD ± ID ± DD (% ± SE)	aOR (95% CI)
Does not fully understand what is being said to him/her	45.4 ± 3.6	27.9 ± 2.0	2.1 (1.5–3.0)*
Overreacts to everything and everyone	27.5 ± 3.1	15.8 ± 1.6	1.9 (1.3–2.9)*
Does not look up when spoken to	25.4 ± 3.3	11.4 ± 1.2	2.6 (1.8–4.0)*
Acts as if others are not there	21.2 ± 2.9	9.6 ± 1.5	2.7 (1.7–4.5)*
Makes little eye contact	26.1 ± 3.1	14.2 ± 1.6	2.1 (1.4–3.3)*
Dislikes physical contact	8.7 ± 1.7	7.5 ± 1.2	1.6 (1.0–2.8)
Does not respond to attempts by others to initiate contact	8.7 ± 1.8	5.1 ± 0.9	1.5 (0.8–2.6)
Shows sudden mood changes	45.1 ± 3.6	26.2 ± 2.0	2.3 (1.6–3.3)*
Gets angry quickly	46.0 ± 3.6	29.2 ± 2.0	2.1 (1.5–3.0)*
Does not realize when there is danger	27.9 ± 3.1	15.6 ± 1.6	2.2 (1.5–3.4)*
Barely knows the difference between strangers and familiar people	18.5 ± 2.6	8.4 ± 1.0	2.4 (1.5–3.8)*
Is disobedient	25.5 ± 3.1	14.1 ± 1.6	2.1 (1.4–3.2)*
Panics in new situations or if change occurs	29.6 ± 3.2	15.5 ± 1.4	2.4 (1.6–3.5)*
Remains clammed up in new situations or if change occurs	22.7 ± 3.3	11.0 ± 1.4	2.6 (1.7–4.1)*
Gets lost easily	23.6 ± 2.9	5.3 ± 0.8	5.2 (3.3–8.4)*

**Notes:**

*denotes significance; for selected CSBQ items, the odds of indicating that a characteristic was “certainly true” of their child was compared between those with and without a history of elopement.

### Preventive Measure Use

Physical barriers to elopement were more commonly used than electronic tracking devices (Table 7). Across all three condition groups, respondents who indicated that their child had eloped within the previous year were significantly more likely to report use of physical or electronic preventive measures (Table 7). Logistic regression analyses (Table 8) indicated that the odds of preventive measure use were significantly higher for the ASD+ID/DD group than for either the ASD-only group (OR: 2.4, 95% CI: 1.5–4.0) or the ID/DD-only group (OR: 3.0, 95% CI: 2.1–4.3). These differences remained significant even after controlling for elopement history.

**Table 7:** Preventive Measure Use by Condition Group.

Condition Group	Physical Barriers (% ± SE)	Electronic Measures (% ± SE)	Any Prevention Strategy: Wanderers (% ± SE)	Any Prevention Strategy: Non-Wanderers (% ± SE)	OR (95% CI)
ASD-only	19.4 ± 3.4	2.7 ± 1.3	34.0 ± 7.3	12.8 ± 3.7	3.5 (1.4–8.8)*
ASD + ID/DD	36.9 ± 2.8	3.5 ± 0.8	54.7 ± 5.2	28.5 ± 3.3	3.0 (1.8–5.1)*
ID/DD-only	15.5 ± 2.0	1.5 ± 0.5	45.7 ± 5.9	8.0 ± 1.1	9.6 (5.6–16.6)*

**Notes:**

*denotes significance. Logistic regression analyses were used to assess the association between elopement history and preventive measure use.

**Table 8:** Between-group Comparisons of the Odds of Any Preventive Measure Use.

Condition Groups	OR (95% CI)	aOR^¥^ (95% CI)
ASD-only vs. ID/DD-only (ref)	1.2 (0.7–2.1)	1.0 (0.6–1.8)
ASD+ID/DD vs. ID/DD-only (ref)	3.0* (2.1–4.3)	2.8* (1.9–4.1)
ASD+ID/DD vs. ASD-only (ref)	2.4* (1.5–4.0)	2.7* (1.5–4.9)

**Notes:**

*denotes significance;

^**¥**^ adjusted for wandering history;

ref = reference group.

## Discussion

Elopement places children with developmental disabilities at risk of serious injury or even death. Despite its clear relevance to the safety and well-being of this population, there has been a paucity of research on this subject to date. This represents the first study to report the prevalence and correlates of wandering in a nationally representative sample of school-age children in the United States with developmental conditions.

In the present study, the prevalence and correlates of elopement were examined for three carefully-chosen, mutually-exclusive condition groups: ASD-only, without ID or DD; ASD with ID and/or DD; ID and/or DD without ASD. In an unpublished, preliminary analysis of the Pathways data, Rice et. al., of the CDC, took a different approach by comparing elopement rates across four groups: ASD with or without DD; ASD with ID; ID without ASD; DD without ID or ASD [9]. The key difference between these approaches is in the grouping of children with ASD and DD. Whereas Rice et. al.’s approach combined the ASD-only and ASD+DD children into a single group and compared them to the ASD+ID children, our approach grouped together the ASD+DD and ASD+ID children and compared them to those with ASD-only. This distinction is important, given that all diagnostic classifications in the Pathways survey were based on parent report, and that no attempt was made to independently assess cognitive functioning. Since the Pathways survey specifically defined DD as “a developmental delay that affects [the child’s] ability to learn,” it is probable that some of the children that were classified as having ASD+DD (without ID) actually had ID or significant cognitive impairment. Previous studies that utilized sources other than parent report to assess the prevalence of cognitive impairment among children with ASDs have concluded that up to 70% of individuals on the autism spectrum may be affected by ID [10, 11]. A landmark epidemiological study that directly assessed the majority of children identified with ASDs in a U.S. township found that 49% had ID [12]. The prevalence of ID was higher, at 64%, in a population-based study of 987 children with ASD in a major U.S. metropolitan area [13]. A series of other multi-site, population-based studies that utilized medical and educational records found that 31–45% of children with ASDs had an IQ of 70 or less, with an additional 23–24% meeting the criteria for “borderline” ID [14–17]. These estimates are substantially higher than the parent-reported prevalence of ID among children with ASD (303/1420; 21%) in the Pathways sample. By contrast, 65% (924/1420) of the ASD cohort had cognitive impairment when defined as parent report of ID and/or DD. This estimate more closely aligns with the aforementioned figures and supports the possibility that some of the children that were classified as having DD would actually have met the criteria for an ID diagnosis. Thus, Rice et. al.’s inclusion of children with DD in the same group as those with ASD-only may have contaminated that sub-group with respect to cognitive impairment. The present analysis was designed with the aim of more clearly delineating the relationship between elopement and cognitive or learning difficulties.

Among all children in the Pathways sample identified as having any current developmental condition (ASD, ID, and/or DD), the weighted prevalence of elopement within the previous year was 26.7%. Within the condition-based sub-groups that were examined, elopement rates ranged from 22.7% to 34.6%. All of these prevalence estimates are lower than that reported by Anderson et. al, who found that just under half (49%) of their sample of 1,218 children with ASDs had a history of wandering [5]. Methodological considerations likely underlie much of this discrepancy. First, whereas Anderson et. al. asked parents to report any incidents of wandering since age 4, the Pathways survey inquired only about elopement within the previous year. Second, given that Anderson et. al. found that elopement attempts peaked at age 5.4 years, the absence of 4- and 5-year-olds in the Pathways sample also likely contributed to the lower rate of elopement. Thus, a potential weakness of the Pathways survey and our analysis of the data is that the age restrictions may have led to underestimation of the true prevalence of elopement in this population. Nonetheless, compared to Anderson et. al.’s study, the present analysis has several major strengths, including the use of a larger cohort that was not subject to recruitment bias. More importantly, because of the CDC’s sampling approach and statistical weighting of the data from the Pathways survey, the findings are nationally representative of school-age children with ASD and/or ID/DD in the United States. Although little is known about the prevalence of elopement in the general pediatric population, Anderson et. al. found that just 13% of the unaffected siblings of children with ASDs had ever eloped since age 4. Collectively, these findings suggest that elopement is a common problem among U.S. children with developmental disabilities, and that this population is disproportionately affected by this issue compared to their typically-developing peers.

Examination of the clinical correlates of elopement suggested that ASD symptoms are significantly associated with the tendency to wander. Although the prevalence of elopement was found to be similar for the ASD-only and the ASD+ID/DD groups, both groups showed significantly higher odds of elopement than the ID/DD-only group. The difference between the ASD+ID/DD group and the ID/DD-only group remained significant even after controlling for demographic factors. Thus, among those with ID/DD, the presence of ASD was associated with increased odds of elopement—but among those with ASD, the presence of ID/DD was not. Analysis of CSBQ responses showed that elopement was associated not only with the categorical condition of ASD, but also with the degree of ASD symptomatology. The CSBQ has been found to be a useful tool for describing the pattern and severity of behaviors associated with the ASD spectrum, including milder manifestations [7, 8]. Across all three condition groups in the Pathways sample, wanderers scored significantly higher than non-wanderers on CSBQ subscales, indicating that the wanderers tended to have more severe and/or a greater number of ASD symptoms. Notably, this was true even for children in the ID/DD-only group that did not have ASD, according to parent report. Further studies are needed to explore the apparent association between ASD symptoms and wandering behaviors. A better understanding of the causes of elopement in this population may aid in the development of strategies for addressing this problem.

The use of wandering prevention measures—including electronic monitoring devices and/or physical barriers such as gates—was common in this sample, especially among the families of children who had eloped within the previous 12 months. Wandering prevention measures were more frequently used in the ASD+ID/DD group than in either the ASD-only or the ID/DD-only groups, even after controlling for elopement history. Due to the cross sectional nature of this survey, it was not possible to ascertain whether preventive measures were successful in reducing elopement rates in this sample. More research is needed to identify effective methods of preventing elopement in this group of children. However, given that even the best preventive measures are unlikely to avert all cases of elopement, it is equally important that strategies for recovering eloped children be devised. Unfortunately, our analyses indicated that many behavioral characteristics that might interfere with the prompt recovery of an eloped child were themselves associated with the propensity to wander. Compared to those without a history of elopement, children who had wandered within the previous year were more likely to anger quickly and lose their tempers; to be easily frightened; to over-react to the presence of others; to lack awareness of dangerous situations; to be unable to distinguish between strangers and familiar people; and to panic or clam up in new situations. These findings highlight the need to provide first responders with adequate education and preparation for encounters with developmentally disabled children who have eloped. Information about the clinical and behavioral characteristics of children who wander may be used to guide such training efforts.

## Conclusions

Overall, our findings demonstrate that elopement is a pervasive problem in the U.S. population of children with developmental disabilities. Addressing this complex issue will likely require multidisciplinary collaboration among researchers, clinicians, advocates, parents, educators, first responders, and others. Ultimately, it is hoped that a better understanding of the prevalence and correlates of elopement will help to reduce the morbidity and mortality associated with this common phenomenon with potentially tragic consequences.
